# Characteristics and prognosis of patients with pathogenic microorganism-positive sepsis AKI from ICU: a retrospective cohort study

**DOI:** 10.3389/fcimb.2025.1509180

**Published:** 2025-05-15

**Authors:** Panpan Jin, Xihan Meng, Chao Yu, Cong Zhou

**Affiliations:** ^1^ Department of Nephrology and Immunology, Kaifeng Central Hospital, Kaifeng, Henan, China; ^2^ Zhongshan Clinical College, Dalian University, Dalian, China; ^3^ Department of Critical Care Medicine, The First Affiliated Hospital of Anhui Medical University, Hefei, China; ^4^ Department of Critical Care Medicine, Peking University Shenzhen Hospital, Shenzhen, China

**Keywords:** positive for pathogenic microorganisms, sepsis, acute kidney injury (AKI), prognosis, SAPS III, risk factors

## Abstract

**Background:**

Sepsis-associated acute kidney injury (SA-AKI) carries a disproportionately high morbidity and mortality rate. While the synergism between dysregulated host response and renal vulnerability is increasingly recognized, the multifactorial drivers of poor prognosis remain poorly defined. The purpose of this study was to investigate the prognosis and clinical characteristics of patients with pathogenic microorganism-positive SA-AKI.

**Method:**

Using a retrospective analysis approach, we extracted populations from the Medical Information Mart for Intensive Care IV (MIMIC-IV) database that fulfilled the diagnostic criteria for confirmed sepsis with microbiological evidence of pathogenic organisms, and patients were divided into two cohorts according to with or without AKI. The severity of the disease in the two groups was collected for evaluation, and the clinical indicators and prognostic results of the patients were evaluated. The objective of this study was to explore the risk factors affecting the prognosis of patients with pathogenic microorganism-positive SA-AKI.

**Outcome:**

The hospital mortality rate of AKI in patients with pathogenic microbial-positive sepsis was 18.96%. Further analysis showed that the use of vasoactive drug therapy, high lactate level, SAPS II score, SAPS III score, LODS score, and clinical indicators of prolonged hospital stay were independent risk factors for in-hospital mortality in patients with pathogenic microorganism-positive SA-AKI. Among them, SAPS III score plays an important role in predicting the prognosis of sepsis patients with AKI. Further studies found that lactate level was positively correlated with SAPS II score, SAPS III score, and LODS score.

**Conclusion:**

The use of vasoactive drug therapy, high lactate level, SAPS II score, SAPS III score, and LODS score plays an important role in assessing the prognosis of patients with pathogenic microorganism-positive SA-AKI, and multivariate comprehensive assessment is significant in predicting the prognosis of sepsis AKI patients.

## Introduction

Sepsis, which is a systemic inflammatory response syndrome triggered by infection (Singer et al., 2016), has always been a major challenge in clinical medicine. Acute kidney injury (AKI), as one of the common complications of sepsis, significantly increases patient mortality rates and medical burdens (Tian et al., 2014; White et al., 2023; Zarbock et al., 2023; Legrand et al., 2024).

The mechanism of AKI induced by pathogenic microorganism-positive sepsis is complex, primarily involving microvascular dysfunction, inflammatory responses, and metabolic reorganization. During sepsis, pathogenic microorganisms and their by-products trigger a robust immune response, leading to the release of large amounts of cytokines and inflammatory mediators. This cascade results in microvascular endothelial cell damage, increased vascular permeability, and tissue hypoperfusion (Alobaidi et al., 2015; Balkrishna et al., 2023; Bruno et al., 2023; Danica et al., 2024). At the same time, the inflammatory response triggers oxidative stress, further damaging kidney cells. In addition, sepsis-induced metabolic changes, such as lactate accumulation and azotemia, exacerbate the burden on the kidneys and promote the development of AKI. The combination of these factors makes pathogenic microorganism-positive sepsis an important cause of AKI. However, current research on sepsis AKI focuses on the clinical manifestations, pathogenesis, and prognosis of overall sepsis AKI (Yue et al., 2022; He et al., 2023). Cohort studies with a large prognosis in patients with pathogenic microorganism-positive sepsis AKI are lacking.

The purpose of this study was to deeply analyze the clinical characteristics and prognosis of patients with pathogenic microorganism-positive sepsis complicated with AKI, including clarifying the epidemiological characteristics of these patients, identifying the crucial factors affecting the prognosis, and optimizing the clinical management strategies of sepsis patients with AKI to improve their prognosis.

## Materials and methods

### Population

The study population was obtained from the Medical Information Mart for Intensive Care IV (MIMIC IV) version 2.2 database (Johnson et al., 2023). The MIMIC IV 2.2 release covers people from 2008 to 2019. The database records detailed information such as the patient’s demographic information, laboratory tests, medication status, vital signs, surgical procedures, disease diagnosis, medication management, and follow-up survival status. The MIMIC IV v2.2 was approved by the Massachusetts Institute of Technology (No. 0403000206). In the MIMIC VI version 2.2 database, we included patients who met the diagnostic criteria for sepsis 3.0 (Singer et al., 2016) and were positive for the pathogenic microorganism. AKI was defined according to the Kidney Disease Improving Global Outcomes (KDIGO) criteria (Ricci and Romagnoli, 2018). In addition, all the participants in the study were aged 18 years or older.

### Data collection

In this study, the baseline data of patients, Sequential Organ Failure Assessment (SOFA) score, Logical Evaluation System for Organ Dysfunction (LODS) score, Oxford Acute Severity of Illness Score (OASIS), Simplified Acute Physiology Score (SAPS) II, and SAPS III were recorded at 24 h after admission. In this study, we searched for the worst values of vital signs and laboratory tests at 24 h of admission. The prognosis of patients with vasoactive drugs, continuous renal replacement therapy (CRRT), and inotropic/vasopressor support during hospitalization was recorded, as well as the prognosis of patients such as length of hospital stay, ICU stay, in-hospital mortality, 28-day mortality, and 90-day mortality.

### Statistics

In this study, the continuous variables were skewed and described in the form of quartiles, and the categorical variables were described in the form of percentiles. Continuous variables were compared between the survival group and the non-survival group by Wilcoxon rank. Categorical variables were compared between the survival group and the non-survival group by Fisher’s exact tests. The hospital mortality, 28-day mortality, and 90-day mortality rates of patients treated with vasoactive drugs versus those without vasoactive drugs were compared by Kaplan–Meier (KM) curves. Multivariate COX regression analysis was used to explore the independent risk factors for in-hospital mortality in sepsis patients with AKI. The area under the ROC curve was used to explore the predictive power of risk factors for in-hospital mortality, 28-day mortality, and 90-day mortality rates in patients with sepsis AKI. Pearson’s test was used for correlation analysis. In this study, indicators with missing values greater than 20% were deleted, and indicators with missing values less than 20% were processed by multiple imputation. The statistical analysis was performed by R software, and *P* < 0.05 was considered to be statistically significant.

## Outcome

### Baseline data of patients with pathogenic microorganism-positive sepsis AKI

A total of 19,658 patients met the diagnostic criteria for Sepsis 3.0. Among these, 9085 patients with sepsis were diagnosed with AKI, and of these 9085 cases, 1820 were positive for pathogenic microorganisms. **Table 1** presents the baseline data of sepsis patients with pathogenic microorganism-positive sepsis with AKI. A total of 1820 patients with pathogenic microorganism-positive sepsis AKI were divided into the survival group (1475 cases) and the non-survival group (345 cases) according to the hospital mortality rate. **Table 1** shows that compared with the patients in the survival group, the patients in the non-survival group were older, had higher levels of Charlson score, white blood cell, PT, APTT, INR, bun, and lactate, as well as faster heart and respiratory rates; additionally, they exhibited lower systolic blood pressure, hemoglobin, platelets, and PH values.

**Table 1:** Baseline of sepsis patients with AKI.

Characteristic	Survival group (n=1475)	Non-survival group (n=345)	*P*
Age, years	69.11 [58.61, 79.66]	73.09 [61.91, 81.02]	0.003
Sex, n (%)			
Male	861 (58.4)	196 (56.8)	0.639
Female	614 (41.6)	149 (43.2)	
Pathogenic microorganisms, n (%)			
*Acinetobacter baumannii*	29 (2.0)	7 (2.0)	1.000
*Klebsiella pneumoniae*	300 (20.3)	50 (14.5)	0.016
*Pseudomonas aeruginosa*	238 (16.1)	30 (8.7)	0.001
*Staphylococcus aureus*	198 (13.4)	30 (8.7)	0.022
*Escherichia coli*	409 (27.7)	60 (17.4)	<0.001
Comorbid conditions, n (%)			
Charlson	6.00 [4.00, 8.00]	6.00 [5.00, 8.00]	0.005
Hypertension	855 (58.0)	171 (49.6)	0.006
Diabetes	644 (43.7)	110 (31.9)	<0.001
Chronic kidney disease	543 (36.8)	108 (31.3)	0.063
Physiology			
Heart rate >100, beats per minute	476 (32.3)	138 (40.0)	0.008
Systolic blood pressure <90, mmHg	315 (21.4)	98 (28.4)	0.006
Diastolic blood pressure <60, mmHg	959 (65.0)	235 (68.1)	0.304
Respiratory rate, beats per minute	22.00 [18.00, 27.00]	23.00 [19.00, 28.00]	0.014
Laboratory tests			
Complete blood count			
White blood cell (×10^9^/L)	12.70 [8.80, 17.70]	14.30 [9.60, 19.75]	0.006
Hemoglobin (g/dL)	9.10 [7.80, 10.60]	8.60 [7.60, 10.30]	0.023
Platelet (×10^9^/L)	172.50 [114.00, 242.00]	150.00 [78.00, 223.00]	<0.001
Coagulation function			
PT (sec)	15.50 [13.20, 18.80]	17.70 [14.20, 23.10]	<0.001
APTT (sec)	35.70 [29.80, 45.20]	44.78 [32.20, 60.10]	<0.001
INR	1.40 [1.20, 1.72]	1.60 [1.30, 2.10]	<0.001
Kidney function			
Creatinine (mg/dL)	1.50 [1.10, 2.50]	1.60 [1.10, 2.60]	0.164
Bun (mg/dL)	31.00 [20.00, 50.00]	35.00 [23.00, 57.00]	0.001
Other laboratory tests			
PH	7.38 [7.33, 7.43]	7.37 [7.30, 7.42]	<0.001
Lactate (mmol/L)	1.90 [1.30, 2.50]	2.50 [1.60, 4.00]	<0.001

APPT, activated partial thrombin time; BUN, blood urea nitrogen; INR, international normalized ratio; PT, prothrombin time. *P* < 0.05, statistically significant.

### Outcome of patients with pathogenic microorganism-positive sepsis AKI

The results of **Table 2** show that compared with the survival group, more patients in the non-survival group were treated with vasoactive drugs and CRRT, had longer ICU stays and hospital stays, and had higher SOFA, SAPS II, SAPS III, LODS, and OASIS scores.

**Table 2:** Outcome of sepsis patients with AKI.

Characteristic	Survival group (n=1475)	Non-survival group (n=345)	*P*
Inotropic/vasopressor support, n (%)	569 (38.6)	218 (63.2)	<0.001
Renal replacement therapy, n (%)	116 (7.9)	38 (11.0)	0.074
ICU length of stay (days)	2.92 [1.58, 6.32]	3.95 [2.01, 8.95]	<0.001
Length of hospital stay (days)	10.93 [5.96, 20.72]	14.16 [8.07, 26.61]	<0.001
Prognostic score			
SOFA score	3.00 [2.00, 5.00]	4.00 [2.00, 6.00]	<0.001
SAPS II score	39.00 [31.00, 48.00]	46.00 [38.00, 57.00]	<0.001
SAPS III score	48.00 [38.00, 60.00]	62.00 [49.00, 80.00]	<0.001
LODS	5.00 [3.00, 7.00]	7.00 [5.00, 10.00]	<0.001
OASIS	31.00 [26.00, 37.00]	36.00 [29.00, 42.00]	<0.001

SAPS, Simplified Acute Physiology Score; SOFA, Sequential Organ Failure Assessment; LODS, Logical Evaluation System for Organ Dysfunction; Sirs, systemic inflammatory response syndrome; OASIS, Oxford Acute Severity of Illness Score; SIRS, Systemic Inflammatory Response Syndrome score. *P* < 0.05, statistically significant.

### KM curves of 28-day and 90-day mortality of vasoactive drugs in patients with pathogenic microorganism-positive sepsis AKI

**Figure 1:**
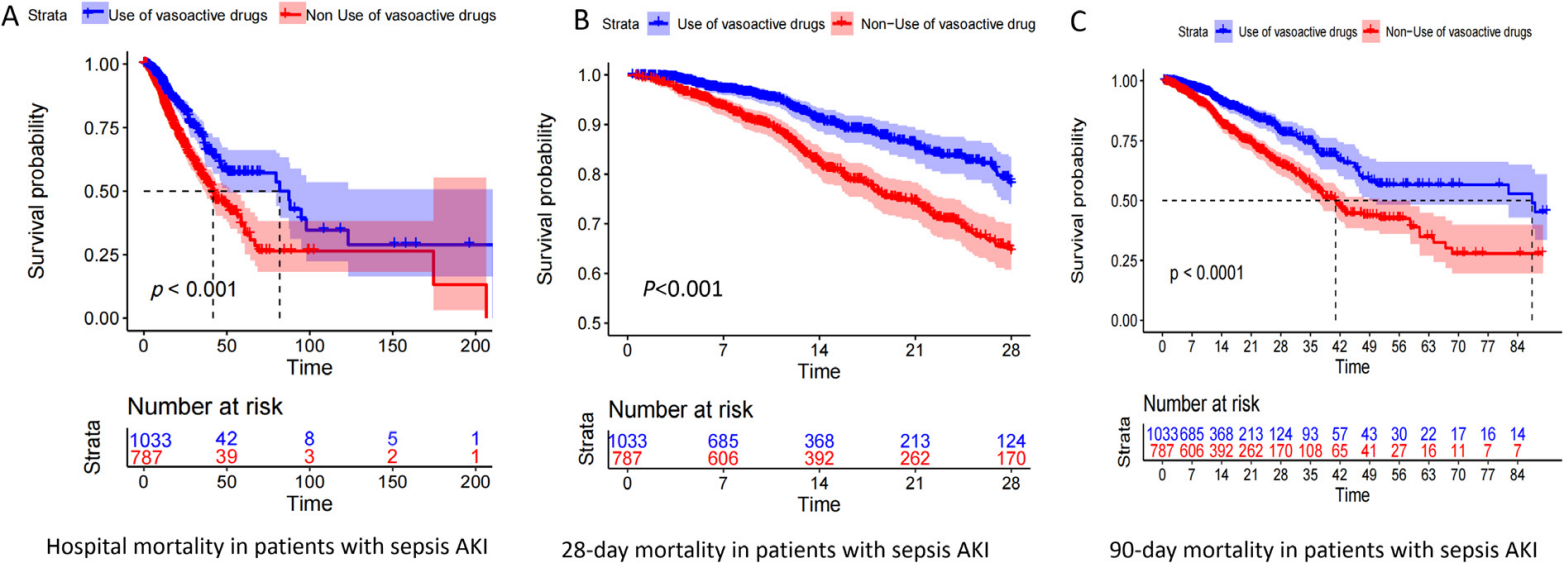
Kaplan–Meier (KM) curve of pathogenic microorganism-positive sepsis AKI prognosis. Panels **(A–C)** show the KM curves of in-hospital mortality, 28-day mortality, and 90-day mortality in patients who were positive for pathogenic microorganism sepsis for AKI, respectively, treated with and without vasoactive drugs.

According to **Table 2**, vasoactive drug use is a risk factor for in-hospital mortality in patients with pathogenic microorganism-positive sepsis and AKI. The results of **Figure 1** show that the 28-day and 90-day mortality rates of patients treated with vasoactive drugs were significantly higher than those of patients treated with non-vasoactive drugs.

### Multivariate COX analysis of in-hospital mortality in patients with pathogenic microorganism-positive sepsis AKI

According to the results of the study in **Tables 1** and **2**, multivariate COX analysis was performed in patients with pathogenic microorganism-positive sepsis AKI. **Table 3** shows the results of the study, indicating that lactate level, use of vasoactive drugs, SAPS II score, SAPS III score, LODS score, and length of hospital stay were independent risk factors for in-hospital mortality in patients with pathogenic microorganism-positive sepsis AKI.

**Table 3:** Multivariate COX regression analysis for sepsis patients with AKI.

Characteristic	HR	95% CI		*P*
		Lower	Upper	
Age	1.005	0.997	1.013	0.236
Charlson	1.030	0.986	1.076	0.186
Heart rate >100, beats per minute	1.092	0.865	1.378	0.459
Systolic blood pressure <90, mmHg	1.093	0.848	1.408	0.493
Respiratory rate	1.009	0.993	1.024	0.269
White blood cell	1.001	0.992	1.009	0.875
Hemoglobin	1.013	0.960	1.069	0.630
Platelet	1.000	0.999	1.001	0.942
PT	1.002	0.994	1.010	0.705
INR	0.970	0.892	1.056	0.483
APTT	1.002	0.999	1.005	0.230
BUN	1.000	0.996	1.004	0.858
PH	0.479	0.168	1.361	0.167
Lactate	1.045	1.010	1.080	0.011
CRRT	0.965	0.672	1.387	0.849
Use of vasoactive drugs	1.605	1.267	2.033	<0.001
SOFA	0.990	0.949	1.032	0.623
SAPS II	1.008	1.001	1.015	0.032
SAPS III	1.012	1.007	1.017	<0.001
LODS	1.047	1.012	1.083	0.007
OASIS	1.001	0.989	1.013	0.850
Los_icu	1.009	0.990	1.029	0.358
Los_hospital	0.027	0.018	0.039	<0.001

APPT, activated partial thrombin time; BUN, blood urea nitrogen; INR, international normalized ratio; PT, prothrombin time; SOFA, Sequential Organ Failure Assessment score; CRRT, continuous renal replacement therapy; SAPS, Simplified Acute Physiology Score; LODS, Logistic Organ Dysfunction System score; OASIS, Oxford Acute Severity of Illness Score. *P* < 0.05, statistically significant.

### ROC curves, specificity, and sensitivity of pathogenic microorganism-positive sepsis AKI hospitalization for predicting prognosis

**Figure 2:**
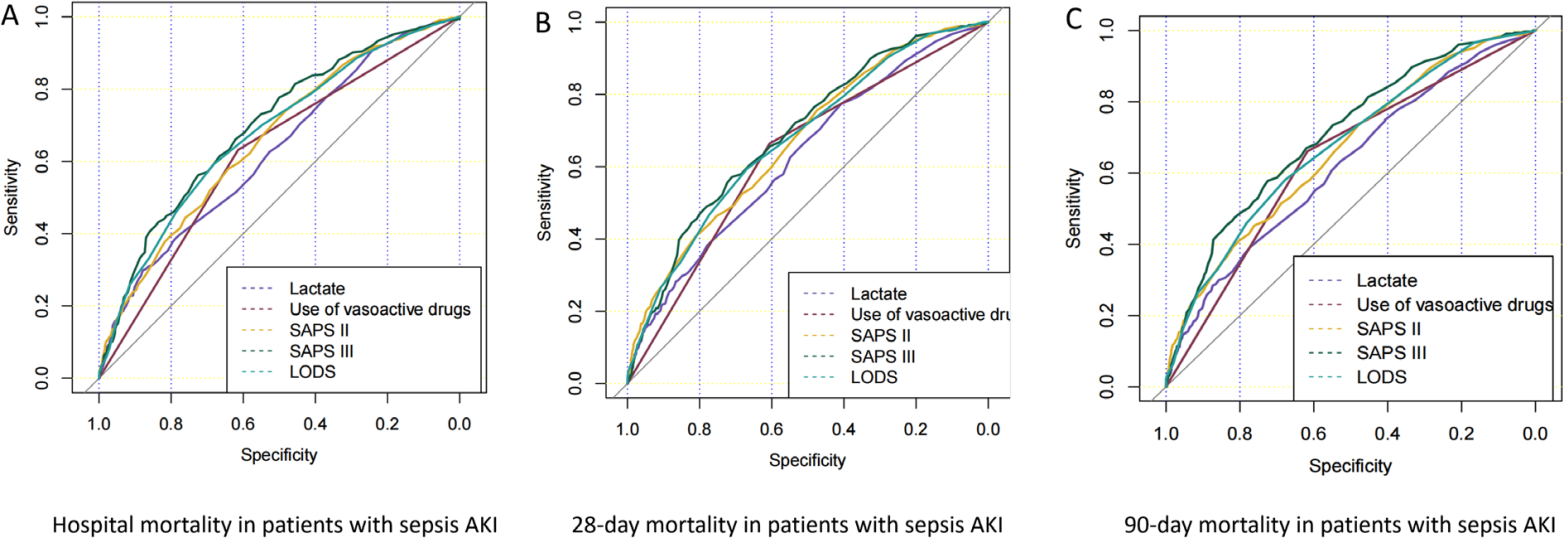
ROC curves of prognostic risk factors for pathogenic microorganism-positive sepsis AKI. Panels **(A–C)** show the ROC curves of in-hospital mortality, 28-day mortality, and 90-day mortality in patients who were positive for pathogenic microorganism sepsis for AKI.

**Figure 2** shows the ROC curves of biomarkers for predicting in-hospital mortality (**Figure 2A**), 28-day mortality (**Figure 2B**), and 90-day mortality (**Figure 2C**) in pathogenic microbial-positive sepsis AKI patients. The results of **Figure 2** and **Table 4** show that SAPS III score had the highest AUC and specificity in predicting in-hospital mortality (0.697 and 0.725), 28-day mortality (0.691and 0.710), and 90-day mortality (0.707 and 0.725) in pathogenic microorganism-positive sepsis AKI and that SAPS II score had the highest sensitivity in predicting in-hospital mortality (0.739), 28-day mortality (0.757), and 90-day mortality (0.734) in pathogenic microorganism-positive sepsis AKI.

**Table 4:** Prognostic indicators for the prognosis of sepsis patients with AKI.

Indicators	AUC	*P*	Specificity	Sensitivity	PPV	NPV
Hospital mortality						
Lactate	0.628	<0.001	0.879	0.299	0.365	0.843
Use of vasoactive drugs	0.623	<0.001	0.614	0.632	0.277	0.877
SAPS II	0.660	<0.001	0.483	0.739	0.251	0.888
SAPS III	0.697	<0.001	0.725	0.562	0.323	0.876
LODS	0.676	<0.001	0.683	0.591	0.304	0.877
28-day mortality						
Lactate	0.627	<0.001	0.406	0.776	0.178	0.916
Use of vasoactive drugs	0.635	<0.001	0.606	0.664	0.219	0.916
SAPS II	0.671	<0.001	0.474	0.757	0.193	0.922
SAPS III	0.691	<0.001	0.710	0.571	0.247	0.909
LODS	0.676	<0.001	0.668	0.595	0.229	0.909
90-day mortality						
Lactate	0.621	<0.001	0.77	0.394	0.273	0.853
Use of vasoactive drugs	0.639	<0.001	0.618	0.661	0.274	0.893
SAPS II	0.663	<0.001	0.480	0.734	0.236	0.892
SAPS III	0.707	<0.001	0.725	0.578	0.315	0.887
LODS	0.675	<0.001	0.677	0.581	0.283	0.881

SAPS, Simplified Acute Physiology Score; LODS, Logistic Organ Dysfunction System score; OASIS, Oxford Acute Severity of Illness Score; PPV, positive predictive value; NPV, negative predictive value.

### Correlation analysis between lactate level and SAPS II, SAPS III, and LODS scores

**Figure 3:**
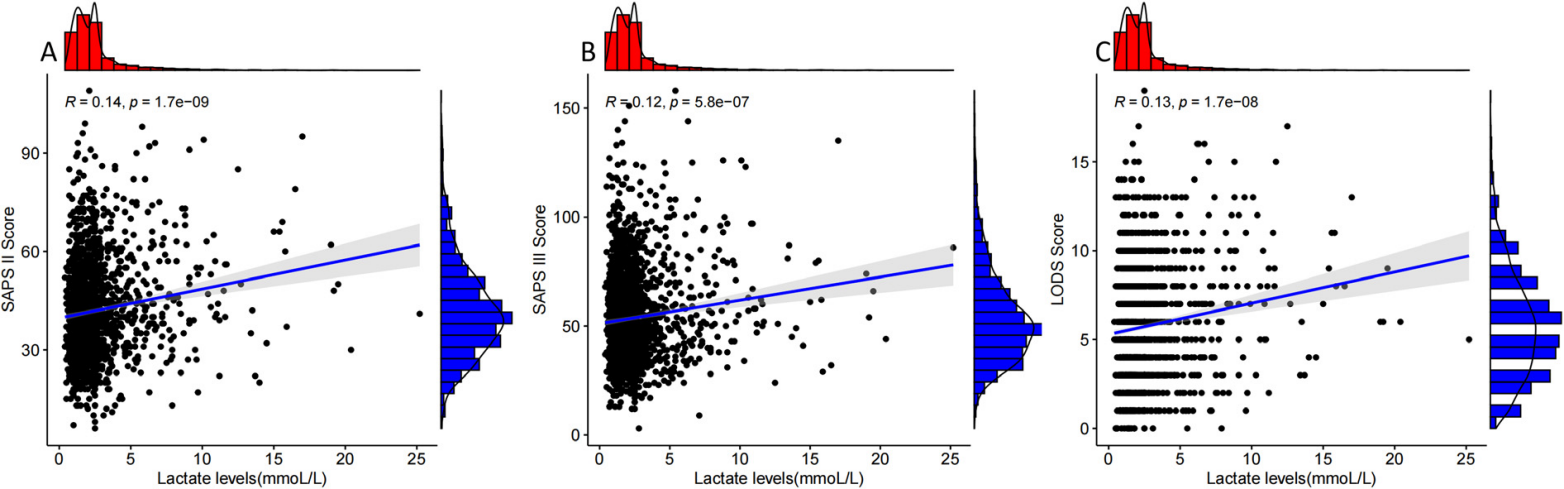
Correlation analysis of lactate levels with SAPS II, SAPS III, and LODS scores. Panels **(A–C)** show the correlation analysis curves of lactate levels with SAPS II, SAPS III, and LODS scores in patients who were positive for pathogenic microorganism sepsis for AKI.

The results in **Figure 3** show that lactate level was positively correlated with SAPS II (R=0.14), SAPS III (R=0.12), and LODS scores (R=0.13).

## Discussion

This study demonstrated that the mortality rate of patients with pathogenic microorganism-positive sepsis-associated AKI was still high. The use of vasoactive drug therapy; elevated lactate levels; higher SAPS II, SAPS III, and LODS scores; and prolonged hospital stays were identified as independent risk factors for in-hospital mortality in these patients. Notably, the SAPS III score emerged as a valuable tool for assessing the prognosis of patients with pathogenic microorganism-positive sepsis-associated AKI. These findings enhance our understanding of the disease and provide valuable insights for clinical diagnosis and treatment.

Sepsis-associated AKI has a high incidence and mortality; previous studies have shown that the mortality rate in patients with sepsis-associated AKI is approximately 30% (Bai et al., 2020; Atreya et al., 2023; Fan Z et al., 2023; Baeseman et al., 2024). The results of this study indicate that the in-hospital mortality rate of patients with pathogenic microorganism-positive sepsis-associated AKI is as high as approximately 20%. These findings align with and further support those of several previous studies, confirming that patients with sepsis-associated AKI have a high mortality rate. Therefore, it is essential to analyze the clinical characteristics of patients with AKI caused by pathogenic microorganism-positive sepsis, identify key factors influencing disease progression, and evaluate their impact on patient prognosis. Such efforts will provide a scientific basis for the early identification of high-risk patients, optimization of treatment strategies, and improvement of clinical outcomes.

This study identified the use of vasoactive drug therapy; elevated lactate levels; higher SAPS II, SAPS III, and LODS scores; and prolonged hospital stays as independent risk factors for in-hospital mortality in patients with pathogenic microorganism-positive sepsis-associated AKI. The use of vasoactive drugs in these patients highlights circulatory failure as a significant contributor to mortality. Circulatory collapse can further exacerbate multiorgan dysfunction, particularly renal injury (Rivers et al., 2001; Holmes, 2003; Morrell et al., 2009; Zhao L et al., 2022; Hasegawa et al., 2023). Therefore, for patients with sepsis-associated AKI treated with vasoactive drugs, it is crucial to aggressively address the causes of circulatory failure, maintain adequate renal and other organ perfusion, and implement strategies to reduce mortality.

The higher the SAPS II score, SAPS III score, and LODS score, the higher the mortality rate of patients with AKI sepsis; these scores are important for assessing the prognosis of critically ill patients and patients with sepsis (Freire et al., 2002; Lima et al., 2005; Rezende et al., 2008; Jihane et al., 2012). This study found that the above scores still play an important role in assessing the prognosis of patients with pathogenic microorganism-positive sepsis AKI, especially SAPS III; therefore, patients with pathogenic microorganism-positive sepsis AKI should be closely monitored for changes in the above score levels to predict the risk of poor prognosis in patients.

In addition, the higher the blood lactate level, the higher the SAPS II, SAPS III, and LODS scores of patients with AKI with sepsis, indicating that the more severe the disease, the higher the mortality rate of patients with AKI sepsis. Lactate reflects tissue hypoperfusion, indicating underperfusion of patients, and multiple studies have shown a significant correlation between lactate and mortality in severe patients (Jones et al., 2010; Woolum et al., 2018; Zhang et al., 2024), and the results of this study further support the previous study. Therefore, patients with high lactate levels of pathogenic microorganism-positive sepsis should be treated with aggressive anti-infection, fluid resuscitation, refined fluid management, and vasoactive drugs to maintain the perfusion level of various tissues, especially the kidneys.

Our study provides important insights into the clinical characteristics and outcomes of patients with pathogen-positive sepsis-associated AKI, which have several implications for clinical practice: the identification of specific pathogenic microorganisms and their association with AKI severity and outcomes can aid clinicians in early risk stratification. This knowledge may help in prioritizing high-risk patients for more intensive monitoring and intervention; the factors associated with poor outcomes identified in our study can serve as prognostic markers. This information can guide discussions with patients and families regarding expected outcomes and informed decision making in clinical care.

### Limitations

Due to the limited sample size and geographical characteristics, this study may not fully reflect the characteristics of all patients with pathogenic microorganism-positive sepsis-associated AKI. In the future, the sample scope should be expanded, multicenter and prospective studies should be conducted, and the risk factors affecting the occurrence, development, and prognosis of AKI should be deeply explored to optimize the clinical diagnosis and treatment strategy.

## Conclusion

This study demonstrated that patients with pathogenic microorganism-positive sepsis-associated AKI had higher SAPS III scores, SAPS II scores, LODS scores, and lactate levels, which can serve as important indicators for prognostic evaluation. These findings enhance our understanding of the disease and provide valuable insights for clinical diagnosis and treatment.

## Data Availability

Publicly available datasets were analyzed in this study. The data in this study were derived from the public database MIMIC IV, from which raw data are available: https://physionet.org/content/mimiciv/2.0/.
